# Cortical microtubule orientation in *Arabidopsis thaliana* root meristematic zone depends on cell division and requires severing by katanin

**DOI:** 10.1186/s40709-018-0082-6

**Published:** 2018-06-15

**Authors:** Emmanuel Panteris, Barbara-Evelin Diannelidis, Ioannis-Dimosthenis S. Adamakis

**Affiliations:** 1 Department of Botany, School of Biology, Aristotle University of Thessaloniki, 541 24 Thessaloniki, Macedonia, Greece; 2 Department of Botany, Faculty of Biology, National and Kapodistrian University of Athens, 157 84 Athens, Greece

**Keywords:** Aphidicolin, *Arabidopsis thaliana*, Cell division, Katanin, Microtubule orientation, Microtubule severing

## Abstract

**Background:**

Transverse cortical microtubule orientation, critical for anisotropic cell expansion, is established in the meristematic root zone. Intending to elucidate the possible prerequisites for this establishment and factors that are involved, microtubule organization was studied in roots of *Arabidopsis thaliana*, wild-type and the p60-katanin mutants *fra2*, *ktn1*-*2* and *lue1*. Transverse cortical microtubule orientation in the meristematic root zone has proven to persist under several regimes inhibiting root elongation. This persistence was attributed to the constant moderate elongation of meristematic cells, prior to mitotic division. Therefore, *A. thaliana* wild-type seedlings were treated with aphidicolin, in order to prevent mitosis and inhibit premitotic cell elongation.

**Results:**

In roots treated with aphidicolin for 12 h, cell divisions still occurred and microtubules were transverse. After 24 and 48 h of treatment, meristematic cell divisions and the prerequisite elongation ceased, while microtubule orientation became random. In meristematic cells of the p60-katanin mutants, apart from a general transverse microtubule pattern, cortical microtubules with random orientation were observed, also converging at several cortical sites, in contrast to the uniform transverse pattern of wild-type cells.

**Conclusion:**

Taken together, these observations reveal that transverse cortical microtubule orientation in the meristematic zone of *A. thaliana* root is cell division-dependent and requires severing by katanin.

## Background

Axial growth of plant root is achieved by cell proliferation and elongation. New cells are produced in the meristematic zone, prepare for rapid expansion in the transition zone and finally elongate in the fast elongation zone [1]. In all the developmental root zones, anisotropic cell expansion requires transversely oriented cellulose microfibrils in the cell wall, following the arrangement of cortical microtubules [2–5]. Recently, it has been confirmed in *Arabidopsis thaliana* that transverse cortical microtubule orientation is initially established in the meristematic root zone [4, 5]. This orientation is “bequeathed” to the transition and fast elongation zones, as long as cells expand anisotropically parallel to the root axis. In addition, transverse microtubule orientation appears more persistent in the meristematic zone than in the fast elongation zone under experimental conditions that inhibit elongation [4, 5].

To interpret this difference in cortical microtubule orientation persistence, it was suggested that neither genetic nor chemically-induced inhibition of cellulose synthesis and/or conformation may disrupt the cell cycle in meristematic cells. Accordingly, cell expansion prior to cell division perseveres and is a critical factor for establishing and maintaining transverse microtubule orientation in meristematic root cells [4, 5].

In the present study, this hypothesis was challenged. Cortical microtubule organization was studied in wild-type *A. thaliana* primary roots, in which cells stopped dividing after treatment with aphidicolin, a potent inhibitor of DNA replication [6]. In addition, in order to further investigate the factors involved in microtubule orientation, we compared cortical microtubule arrangement in meristematic root cells of the wild-type and of the p60-katanin mutants *fra2* [7], *lue1* [8] and *ktn1/2* [9]. Our findings support that establishment of transverse cortical microtubule orientation in the meristematic zone of *A. thaliana* root is substantially associated to cell division and depends on microtubule severing.

## Methods

Seeds of *A. thaliana*, wild-type (Col-0) and the p60-katanin mutants *fra2*, *lue1* and *ktn1/2*, were surface sterilized and grown on solid agar medium as previously described [10]. All the chemicals and reagents used in this study were purchased from Sigma (Taufkirchen, Germany), Merck (Darmstadt, Germany) and Applichem (Darmstadt, Germany), and all the following steps were carried out at room temperature unless stated otherwise.

Five-day-old wild-type seedlings were transplanted on solid agar medium plates supplemented with 30 μM aphidicolin and further grown for 12, 24 or 48 h. Untreated and aphidicolin-treated wild-type seedlings, as well as untreated mutant seedlings, were prepared for whole-mount *α*-tubulin immunolabeling as follows: whole seedlings were fixed for 1 h in 4% (w/v) paraformaldehyde in PEM (50 mM PIPES, 5 mM EGTA, 5 mM MgSO_4_, pH 6.8) with the addition of 5% (v/v) dimethylsulfoxide (DMSO). After washing in PEM (3 × 10 min), cell walls were digested for 1 h in 2% (w/v) macerozyme R-10 (Duchefa, Haarlem, Netherlands) in PEM. Then, the seedlings were treated with absolute methanol at − 20 °C for 20 min and subsequently extracted with 5% (v/v) DMSO  and  1% (v/v) Triton X-100 for 1 h. Incubations with rat anti-*α*-tubulin (YOL 1/34, AbD Serotec, Kidlington, UK) and FITC-anti-rat (Invitrogen, Carlsbad, CA), both diluted 1:40 in PEM, were carried out sequentially overnight in the dark with a washing intermediate step (3 × 10 min). Finally, after washing in PEM as previously, the seedlings were mounted in a PEM-glycerol mixture (1:2 v/v) supplemented with 0.5% *p*-phenylenediamine as anti-fade agent. For each treatment, 10 primary roots were studied. Some specimens were slightly squashed between the microscope slide and coverslip, to release the cortex cells from the surrounding tissues. The preparations were examined with a Nikon D-Eclipse C1 or a Zeiss LSM780 confocal laser scanning microscope (CLSM), with the appropriate filters for FITC, and micrographs were acquired with each manufacturer’s software.

In the images obtained with the above CLSM systems, angular distribution of cortical microtubules was analyzed and deciphered using the Microfilament Analyzer software (https://www.uantwerpen.be/en/research-groups/bimef/downloads/microfilament-analyzer/). By means of this software the angular distribution of cortical microtubules could be determined and accordingly inferred to their orientation either as “transverse”, at angles of 90° or 270°, “longitudinal” at angles of 0° or 180° or “random” at angles between 0° and 180° as described in [10]. Statistical analysis (ANOVA with Dunnett’s multiple comparison test) of cell length in untreated and aphidicolin-treated roots was performed with GraphPad (San Diego, CA, USA), with significance at *p *< 0.05.

Roots of untreated wild-type, *fra2*, *lue1* and *ktn1/2* seedlings were also prepared for transmission electron microscopy (TEM) as previously described [11]. In brief, root segments comprising the developmental root zones were fixed for 4 h in 3% (v/v) glutaraldehyde in 50 mM sodium cacodylate, pH 7, post-fixed in 1% (w/v) osmium tetroxide for 3 h, dehydrated in an acetone series and embedded in Spurr’s resin. Ultrathin sections (70–90 nm) were double stained with uranyl acetate and lead citrate and observed with a JEOL JEM 1011 TEM. Images were acquired with a Gatan ES500 W camera. Confocal and TEM images were processed with Adobe Photoshop CS2 with only linear settings.

## Results and discussion

**Fig. 1:**
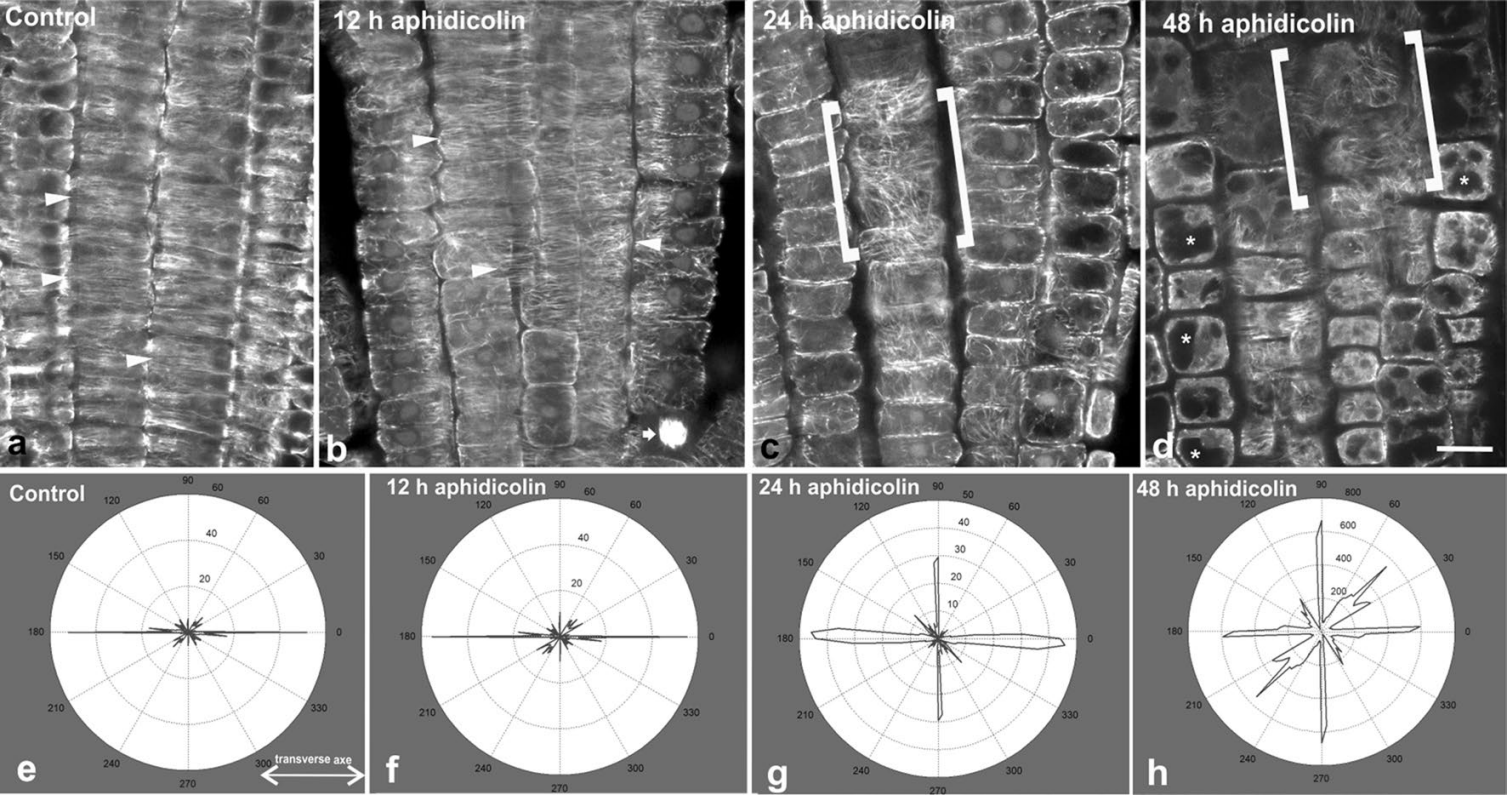
Cortical microtubule orientation in the meristematic zone of untreated (**a**) and aphidicolin-treated (**b**–**d**) roots. Single CLSM sections through the boundary between protoderm and cortex are presented, so that in the center of each figure cortex cells can be observed. In all the images of this work the root tip is oriented towards the bottom of the page. In control root (**a**) prominent transverse orientation of cortical microtubules can be observed in interphase cells (arrowheads). At 12 h of treatment (**b**) transverse microtubule orientation persists (arrowheads), while dividing cells still exist (arrow points to a mitotic cell). After 24 h of treatment (**c**), cell divisions have ceased and cortical microtubules exhibit random orientation (cells within the brackets are cortex cells viewed at external cortical plane), which is also observed after 48 h of treatment (**d**; cells within brackets). In the latter image, vacuolation of meristematic cells in also prominent (asterisks show vacuoles). Representative decipher-graphs of microtubule alignment in meristematic cells of control (**e**), 12 h (**f**), 24 h (**g**) and 48 h (**h**) aphidicolin treatments. Decipher-graphs show that microtubules are transversely oriented (doubled-headed arrow) in the control and after 12 h of aphidicolin treatment (**e**, **f**), while the frequency of longitudinal and randomly oriented microtubules increased upon prolonged treatments (**g**, **h**, respectively). Scale bar 10 μm

As already confirmed, cortical microtubules in the meristematic root zone are transversely oriented, except for those at the external protodermal cell face [4]. At the transition zone mitotic divisions gradually cease, cells attain a cubic shape and start to vacuolate, while their nuclei are positioned at the center [1]. This root zone is still covered by the lateral root cap and its cells typically exhibit transverse microtubule orientation [4]. As a result, to achieve comparable observations, in the meristematic root zone the cells of cortex and endodermis were preferentially studied, because they exhibit fairly transverse cortical microtubules in untreated wild-type roots (Fig. 1a) and their fluorescence is strong and sharp enough, as they reside close to the root surface. Meristematic protodermal cells were avoided, since cortical microtubules in them follow a rather complicated pattern [4, 12], while cells deeper in the root, though encompassing transverse microtubules as well, often exhibit a rather faint signal. In the transition and fast elongation zone, cells of the epidermis were studied.

After 12 h of aphidicolin treatment, cell divisions still occurred (Fig. 1b), though less abundant (Table 1), in the meristematic zone of *A. thaliana* wild-type roots. The mitotic index dropped from 13.22 in untreated roots to 5.93 in roots treated with aphidicolin for 12 h. While in untreated roots 2 preprophase cells and 3 cytokinetic cells were recorded in the transition zone, no dividing cells at all were found in the transition zone of roots treated with aphidicolin for 12 h. Interphase meristematic root cells exhibited transverse cortical microtubule orientation after 12 h of aphidicolin treatment (Fig. 1b).

**Table 1:** Number and percentage of cells at various cell cycle stages, in the meristematic root zone of control and aphidicolin-treated roots

	Control	12 h	24 h	48 h
Interphase	1411 (86.78%)	1872 (94.07%)	1725 (100%)	1648 (100%)
Preprophase/prophase	96 (5.90%)	62 (3.12%)	0 (0%)	0 (0%)
Metaphase/anaphase	25 (1.54%)	15 (0.75%)	0 (0%)	0 (0%)
Telophase/cytokinesis	94 (5.78%)	41 (2.06%)	0 (0%)	0 (0%)

After 24 h of aphidicolin treatment, cell divisions had completely ceased (Table 1) in all the roots that were studied. Cortical microtubules exhibited random orientation in the cells of the meristematic zone (Fig. 1c). The above finding was further confirmed using the Microfilament Analyzer software. It appears that randomly oriented cortical microtubules were amplified over the transverse ones, as aphidicolin treatment duration increased (Fig. 1e–h).

**Fig. 2:**
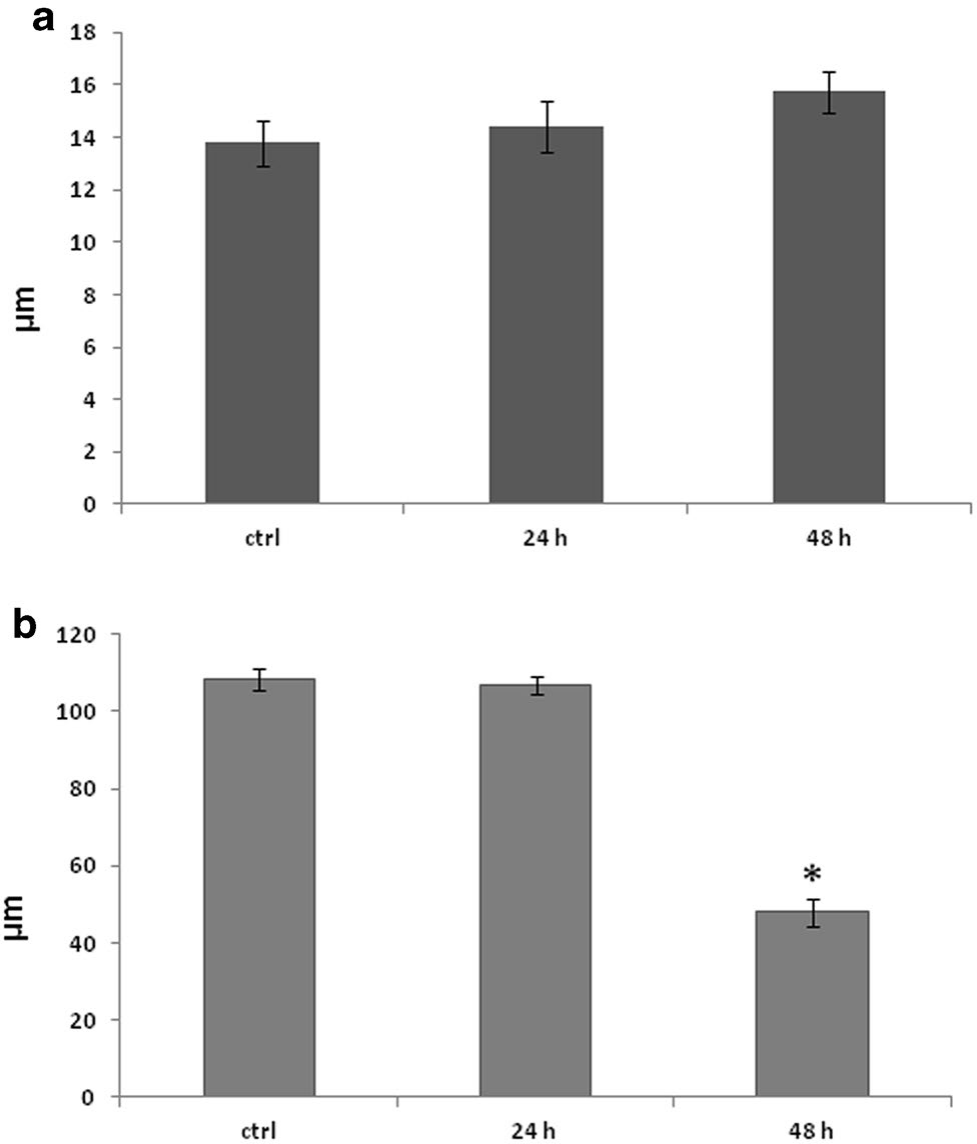
Diagrams depicting the mean length (parallel to the root axis) of meristematic (**a**) and fast elongation (**b**) zone cells in untreated and aphidicolin-treated roots. Error bars represent standard error of the means (n = 300 in **a**, n = 50 in **b**). The asterisk denotes the only statistically significant difference compared to the control (*p *<0.05)

Meristematic cell length (parallel to the root axis) was variable, ranging between 8 and 18 μm (Fig. 2a), resembling that of untreated roots (Fig. 2a; [13]). It appears, therefore, that meristematic cell expansion ceased concomitantly with the aphidicolin-induced cell cycle arrest, resulting in cortical microtubule reorientation. This is similar to the reorientation of cortical microtubules that was observed in fast elongation zone cells, the expansion of which was prematurely stopped [4, 5]. Accordingly, it can be concluded that a major factor for the establishment and maintenance of transverse cortical microtubule orientation in the meristematic zone of *A. thaliana* root is cell division, promoting anisotropic cell expansion parallel to the root axis.

After 48 h of treatment, apart from cessation of cell divisions (Table 1), the cells of the meristematic zone appeared vacuolated and exhibited randomly oriented cortical microtubules (Fig. 1d), while cell length spanned between 9 and 20 μm (Fig. 2a). Vacuolation is typical of meristematic cells under stress [14, 15] and its occurrence can be considered as a side-effect of aphidicolin, which at longer treatment becomes fatal. Elongated nuclei like those reported by Yasuhara and Kitamoto [16] were not observed. However, this may be due to the difference between the cell type studied there (tobacco BY2 cells) and *A. thaliana* root cells.

**Fig. 3:**
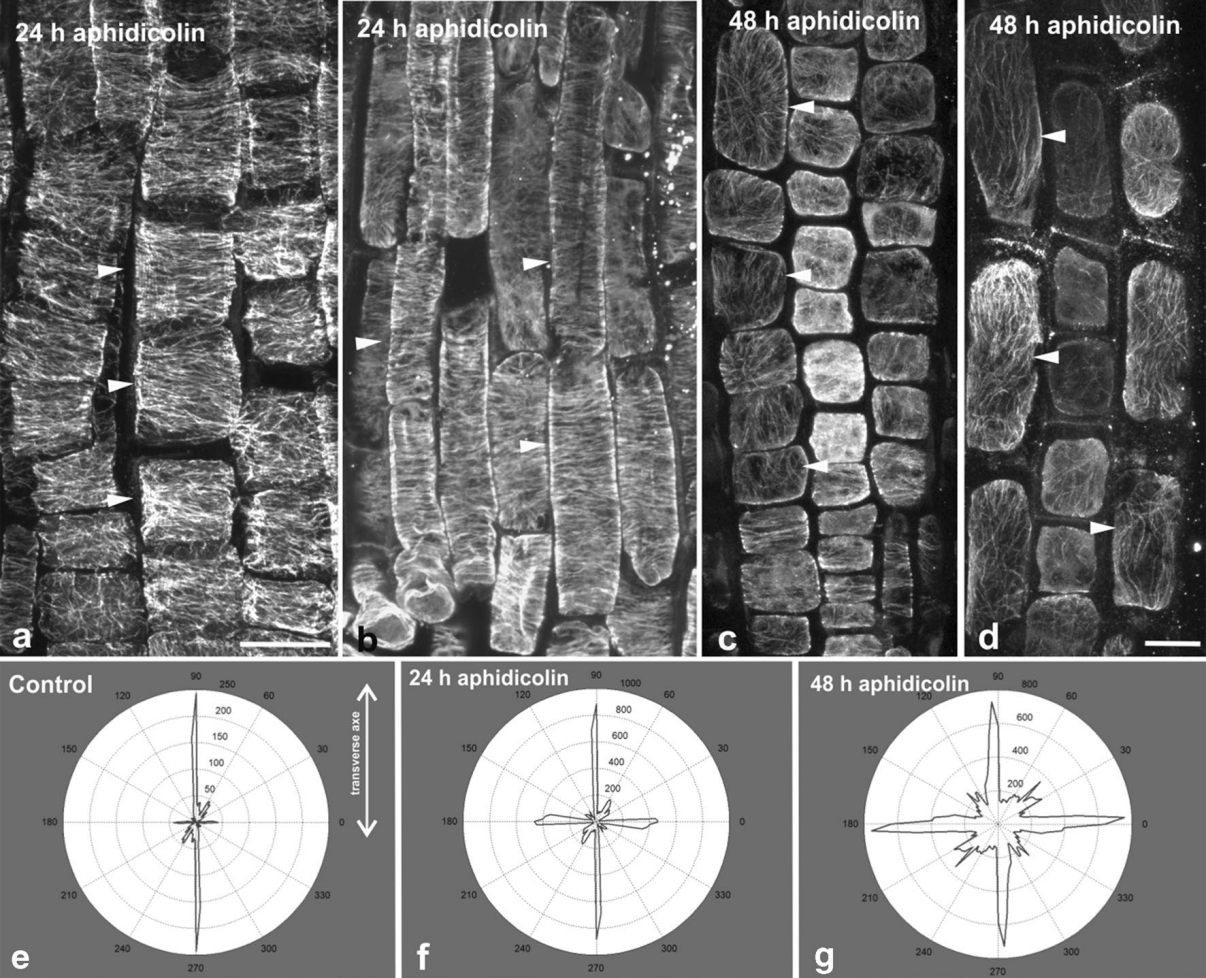
Cortical microtubule orientation at the external face of epidermal cells in the transition (i.e. under the shootward part of the lateral root cap; **a**, **c**) and fast elongation (**b**, **d**) zones of aphidicolin-treated roots. All images are maximum projections of serial CLSM sections. After 24 h of treatment, microtubules are transverse (arrowheads) in the transition (**a**) and fast elongation (**b**) zone, while after 48 h of treatment they appear randomly oriented in both zones (**c**, **d**). Representative decipher-graphs of microtubule alignment in transition and elongation zone cells of control roots (**e**), and after 24 h (**f**) and 48 h (**g**) of aphidicolin treatment. Decipher-graphs show that microtubules are transversely oriented (doubled-headed arrow) in the control and after 24 h of aphidicolin treatment (**e** and **f**, respectively), while the frequency of longitudinal and/or randomly oriented microtubules increased after 48 h of treatment (**g**). Scale bar 10 μm

In roots treated with aphidicolin for 24 h, cortical microtubules in the transition and fast elongation zone cells were still transverse (Fig. 3a, b), in contrast to those of the meristematic zone. Also, the maximum epidermal cell length in the fast elongation zone ranged between 90 μm and 130 μm (Fig. 2b), as in untreated roots [4]. This reveals that arrest of the cell cycle did not affect turgor-driven cell expansion in this zone, thus cortical microtubules retained their transverse orientation to support anisotropic cell expansion as in untreated roots ([4] and references therein). However, after 48 h of treatment, random and/or longitudinal cortical microtubule orientation was obvious in the transition and fast elongation root zones (Fig. 3c, d). The above finding was further confirmed using the Microfilament Analyzer software. It appears that longitudinal and/or randomly oriented cortical microtubules “overthrow” the transverse ones as treatment duration increases (Fig. 3e–g). Maximum cell length in the fast elongation zone was significantly shorter than that of untreated roots, not exceeding 80 μm (Fig. 2b). Apparently, long-term cessation of cell proliferation deprived the transition and fast elongation zones of new cells, resulting in an overall inhibition of root growth. As expected, termination of cell expansion resulted in random cortical microtubule orientation in all the developmental zones of *A. thaliana* root.

**Fig. 4:**
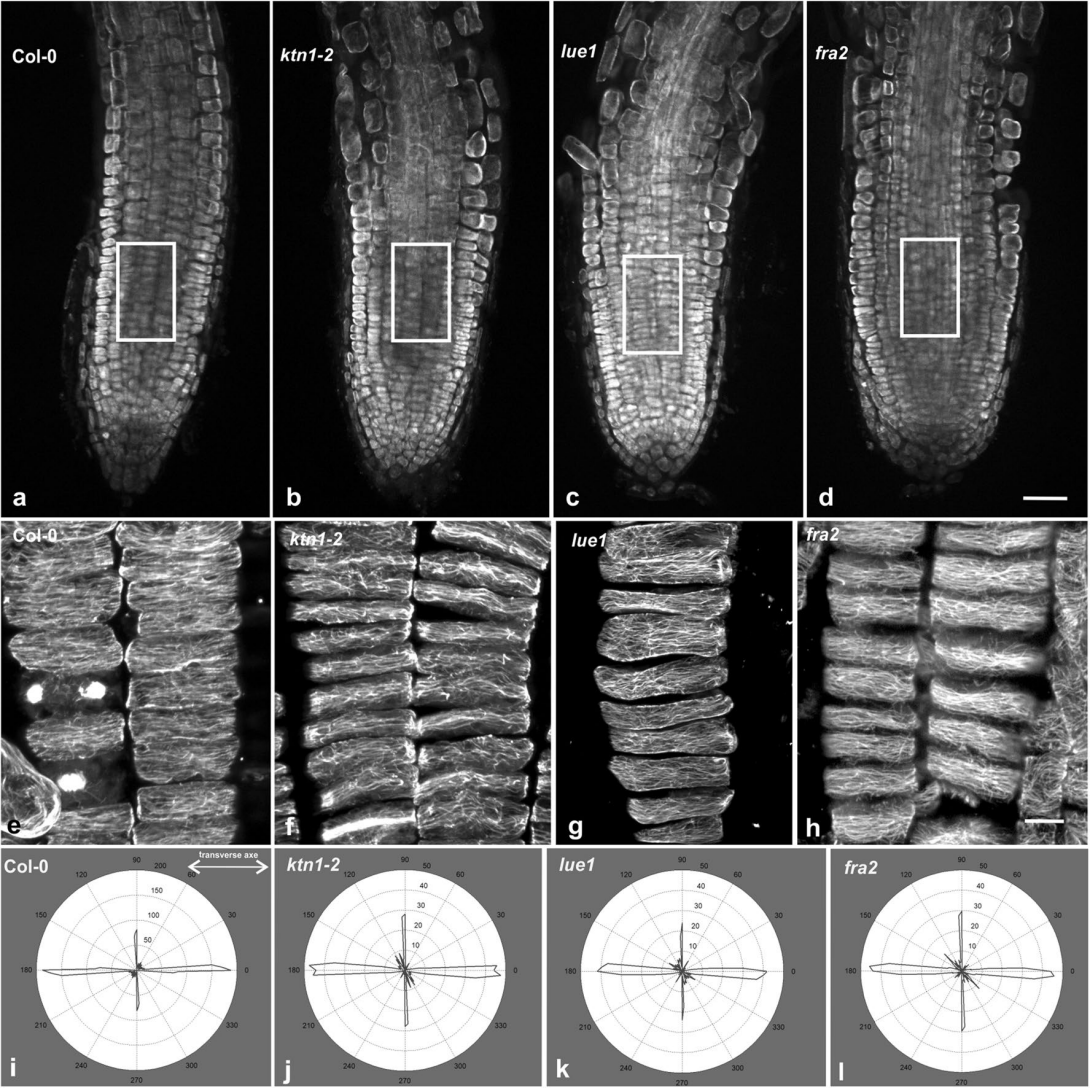
Cortical microtubule orientation in cortex cells of wild-type (**a**, **e**) and p60-katanin mutants (**b**–**d**, **f**–**h**). All high magnification images (**e**–**h**) are maximum projections of serial CLSM sections through the cortical cytoplasm. Cortex cell files, derived from the areas defined by rectangular frames on low magnification images (**a**–**d**), were released after application of gentle pressure on the whole mount root specimens. Fairly transverse cortical microtubules can be observed in Col-0 cells (**e**). In *ktn1*-*2* (**f**), *lue1* (**g**) and *fra2* (**h**), though a general transverse pattern exists, several microtubules exhibit random orientation, while foci of microtubule convergence can be discerned as well. Representative decipher-graphs of microtubule alignment in meristematic cortex cells of Col-0 (**i**), *ktn1*-*2* (**j**), *lue1* (**k**) and *fra2* (**l**). Generally, transverse microtubule orientation prevailed in all the cases (doubled-headed arrow). However, in the katanin mutants an increase in the frequency of longitudinal and/or randomly oriented microtubules was noticed. Scale bars 50 μm (**a**–**d**), 10 μm (**e**–**h**)

**Fig. 5:**
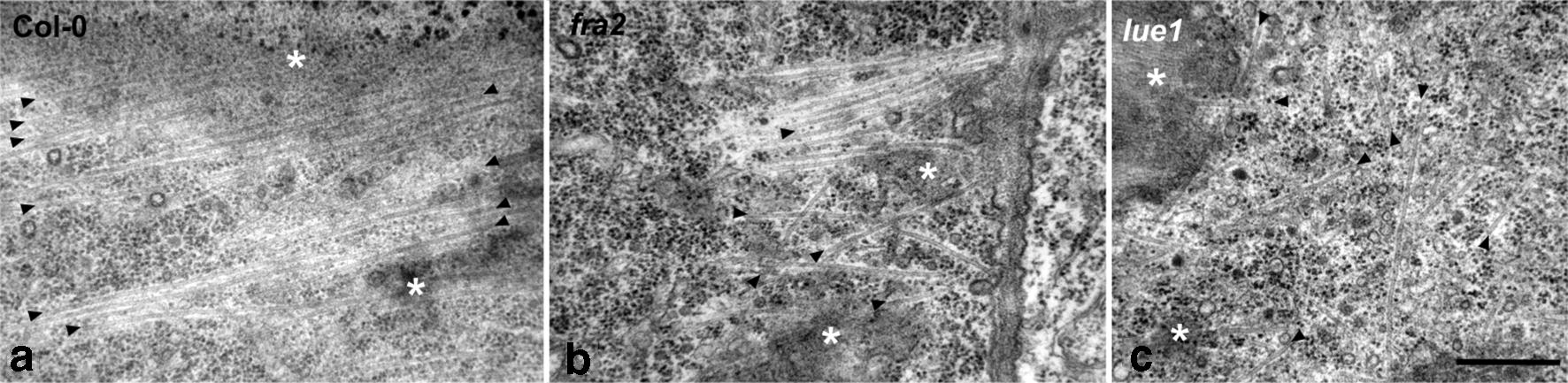
TEM micrographs of cortex cells at tangential sections through the cortical cytoplasm. In the wild-type (**a**), transverse microtubules (arrowheads) uniformly parallel to each other can be observed. In *fra2* (**b**) and *lue1* (**c**), microtubules (arrowheads) follow a random pattern. The asterisks mark grazing sectioned parts of cortex cell walls. Scale bar 400 nm

In all the p60-katanin mutants studied, cortical microtubule arrangement at the external cell face of cortex cells (i.e. cells just beneath the epidermis) of the meristematic root zone was not uniformly transverse as in the wild-type (Fig. 4b–d, f–h; cf. Figure 4a, e). Microtubules with several orientations could be observed by CLSM within a curly-transverse “background” pattern, often interconnecting the transverse microtubules or converging to random foci (Fig. 4f–h), a finding also revealed by microtubule angular image analysis (Fig. 4i–l). The above observation was also confirmed by TEM: while in wild-type root cells cortical microtubules exhibited a uniform parallel arrangement (Fig. 5a), in the mutants their arrangement was non-uniform (Fig. 5b), sometimes appearing chaotic (Fig. 5c). Severing by p60-katanin is pivotal for the organization of microtubule arrays in interphase and mitotic [10, 11, 17–20] cells. However, it has been suggested that, although critical for cortical microtubule orientation in fast elongating cells, severing is not important in interphase meristematic cells, the microtubule pattern of which was described as random [21, 22]. On the other hand, the work of previous authors [23–25], as well as our recent observations [4, 5, the present study], clearly demonstrate that transverse cortical microtubule orientation is established in the meristematic zone and bequeathed in the transition and fast elongation zones. Furthermore, our data support that microtubule severing is required for transverse microtubule orientation since its initiation in the meristematic cells. Importantly, random microtubule orientation in p60-katanin mutants results in decreased cell length in the fast elongation zone [7, 21] as well as in the meristematic zone [11]. Accordingly, microtubule severing is required throughout all the developmental zones of *A. thaliana* root, for both the establishment and maintenance of the transverse pattern that underlies anisotropic expansion. Last but not least, similarly to root meristematic cells, microtubule severing by p60-katanin is also required for proper microtubule organization in shoot meristematic cells [26, 27]. It seems, therefore, that this is a general feature of interphase meristematic cells, important for shoot, root and whole plant development.

## Conclusions

Cell size is a critical factor for the progression of plant cell cycle [28]. Plant cells grow to achieve a size large enough to be divided in two cells, which in turn will grow to divide again. In the root of *A. thaliana* the majority of cell divisions are proliferative, perpendicular to the root axis, especially over the T-divisions [29]. As a result, meristematic cell growth is mainly anisotropic, parallel to the root axis. This moderate expansion, though overlooked [30], is necessary for cell proliferation in the meristematic root zone. Our data provide evidence that the bidirectional interplay between cell expansion and cortical microtubule arrangement, previously shown in fast elongating cells [4, 5], also exists in the meristematic root zone: anisotropic cell expansion is sustained by transversely arranged microtubules, while the latter arrangement requires cell expansion to be established and maintained. Overall, initiation, establishment and maintenance of transverse microtubule orientation in the developmental zones of *A. thaliana* root depend on cell division and require severing by p60-katanin.
